# Effect of a single, oral administration of selenitetriglycerides, at two dose rates, on blood selenium status and haematological and biochemical parameters in Holstein-Friesian calves

**DOI:** 10.1186/s13620-021-00192-4

**Published:** 2021-04-23

**Authors:** Katarzyna Żarczyńska, Przemysław Sobiech, Dawid Tobolski, John F. Mee, Josef Illek

**Affiliations:** 1 Department and Clinic of Internal Diseases, Faculty of Veterinary Medicine, University of Warmia and Mazury, 10-957 Olsztyn, Poland; 2 Animal and Bioscience Research Department, Teagasc, Moorepark Research Centre, Fermoy, Co, P61 C997 Cork, Ireland; 3 Clinical Laboratory for Large Animals, Faculty of Veterinary Medicine, University of Veterinary and Pharmaceutical Sciences, 612 42 Brno, Czech Republic

**Keywords:** Selenium, Selenitetriglycerides, Calves, Glutathione peroxidase, Biochemical parameters, Haematology

## Abstract

**Background:**

Selenitetriglycerides are biologically active, organic forms of selenium formed as a result of the modification of selenic acid and sunflower oil. Studies in rats have shown that they are well absorbed and of low toxicity. There are no published studies on selenitetriglycerides supplementation in calves.

**Results:**

In this study, selenitetriglycerides were administered once orally on the 2nd day of life at a dose of 0.5 or 1 mg Se/kg body weight to each of six Holstein-Friesian calves while six control calves were not supplemented. Blood for determination of selenium concentration, glutathione peroxidase activity, haematological parameters, aspartate aminotransferase, creatine kinase, and lactate dehydrogenase activities and glucose, total protein, albumin, triglycerides, cholesterol, urea, and creatinine concentration was collected before supplementation (day 0) and 1, 2, 5, 10 and 14 days after supplementation. Selenitetriglycerides administration increased (*P* < 0.01) serum selenium concentration in supplemented calves as early as day1, from a mean of 63.4 to 184.22 µg/l in calves receiving selenium at a dose of 0.5 mg/kg BW, and from 63.17 to 200.33 µg/l in calves receiving 1 mg/kg. Serum selenium concentrations remained significantly higher compared to the control group throughout the experiment. Glutathione peroxidase activity was higher in supplemented than control calves, significantly so in animals receiving the 1 mg/kg dose of Se on the 10th and 14th days (*P* < 0.05). There were no significant differences in the haematological and biochemical parameters between the groups.

**Conclusions:**

This experiment showed that supplementation with selenitetriglycerides could significantly improve blood selenium status in calves without adverse effects on haematological or biochemical parameters. These findings are essential prerequisites for future studies on selenitetriglycerides supplementation to manage clinical selenium deficiency in calves.

## Background

Selenium (Se) has diverse biological roles ranging from participation in antioxidant and detoxification processes, through stimulating the proliferation of B cells and the production of IgM and IgG, to antiviral, antibacterial, and anticancer properties [1–3]. Nutritional muscular dystrophy (NMD), also known as white muscle disease, is the most common clinical disorder caused by selenium deficiency in cattle. Young animals with hyposelenosis are also more susceptible to respiratory and gastro-intestinal infections. Additionally, reduced weight gains have been reported in selenium-deficient cattle [4]. The prevalence of clinical selenium deficiency varies widely internationally. It is more commonly observed in pasture-based systems depending on the type of bedrock, pasture selenium content and whether effective selenium supplementation is practiced. Clinical selenium deficiency is less commonly seen in confinement systems where adequately balanced partial (PMR) or total mixed ration (TMR) is fed. Newborn calves should have adequate selenium reserves if the pregnant dam was fed sufficient selenium as the element crosses the placenta. However, where gestational selenium supplementation is inadequate, the newborn calf may be born selenium-deficient [5], and supplementation with this nutrient is required.

Selenitetriglycerides are biologically active, organic forms of selenium in the + 4-oxidation state formed as a result of the modification of selenic acid and sunflower oil. Studies in rats have shown that they are well absorbed after oral, subcutaneous, and intraperitoneal administration. After the absorption of selenium in selenitetriglycerides from the gastrointestinal tract, it is bound by erythrocytes and serum albumin and globulin and transported to tissues [6]. Organic forms of Se have lower toxicity than inorganic forms, specifically, selenitetriglycerides have been shown to have very low toxicity in rats [6].

Due to the presence of many chemical forms of this metalloid, which have different biological properties, including toxicity, the effect of supplementation with selenitetriglycerides may differ between species. For example, while research by Sochocka et al. [7] in healthy Swiss mice showed an increase in the activity of the selenoenzyme glutathione peroxidase (GSH-Px) following selenitetriglycerides administration which was confirmed after selenitetriglycerides administration in people with prostate cancer [8], this response was not found in research conducted on sheep [9]. To date, there have been no equivalent studies in calves.

Given the proven biological activity of selenitetriglycerides in other species, the lack of data in cattle and the potential benefits of such activity in young stock, it was decided to evaluate selenitetriglycerides use in calves. It was hypothesised based partly on studies in other species that a single administration of selenitetriglycerides would increase serum Se concentration and, consequently, blood GSH-Px activity and alter selected haematological and biochemical blood parameters. This study is an essential step to take before evaluating the clinical efficacy of this form of Se in cases of suspected Se deficiency.

## Materials and methods

### Animals, diets, and experimental design

The research was conducted on 18 Holstein-Friesian female calves from one farm located in north-eastern Poland. The experimental calves were randomly selected at one day of age. The calves were from cows in second and third lactation. The cows were housed all year round in a freestall barn and fed a partial mixed ration (PMR) supplemented with a vitamin and mineral premix (9 mg selenium/animal/day) and concentrate at 4 kg/animal throughout the dry period. The animals were randomly allocated to one of three equal (*n* = 6) experimental groups; an unsupplemented control group and two supplemented groups which received selenitetriglycerides at two days of age in a single oral dose of 0.5 and 1 mg Se/kg BW, respectively. These dose rates were chosen based on limited previous experimental studies in rats [6] and sheep [9]. The liquid preparation was administered using a calibrated oral drencher in the morning. The calves were fed 2.5 l of the mother’s colostrum administered by stomach tube 2 h after birth, and another 2 l of the same colostrum 6 h after birth. After a 5-day colostrum period, the seven day old calves received 6–8 l of their own dam’s milk using a teated-bucket, twice a day, and a starter feed in the form of crunch/loose mix from 8 day of life. This feed consisted of micronized corn, soybean meal (non-GMO), micronized barley, wheat gluten, micronized wheat, rapeseed meal, beet molasses, dried alfalfa, calcium carbonate, sodium chloride, monocalcium phosphate, and fodder yeast. The animals were kept in conditions meeting the requirements for farm animals’ welfare in individual pens bedded with straw and had unlimited access to water and hay.

### Sampling and analyses

Blood was collected by venepuncture from the external jugular vein of each calf six times into tubes containing a clot activator (9 ml, Vacuette, Greiner Bio-One, France) for serum analyses of selenium and biochemical parameters, into vacutainers containing K2 EDTA (4 ml, Vacuette, Greiner Bio-One, France) for hematological analysis and into tubes containing lithium heparin (6 ml, Vacuette, Greiner Bio-One, France) for determination of glutathione peroxidase. Samples taken for selenium estimation were stored at -22 C for further determination, the other analyses were conduced withing 4 h after sampling. The first sample was collected on the day of, but before, Se administration (day 0), and subsequent samples were collected 1, 2, 5, 10, and 14 days a.m. after Se administration. Serum Se concentration was determined by hydride generation-flame atomic absorption spectrometry (Unicam 939 Solar Spectrophotometer). The activity of GSH-Px was measured in whole blood by the kinetic method using cumene hydroxide and phosphate buffer in an Epoll 20 analyzer using the Ransel diagnostic kit (Randox Laboratories, Crumlin, UK). The following haematological parameters were determined in whole blood samples: white blood cell count (WBC), red blood cell count (RBC), hemoglobin concentration (HGB), hematocrit (HCT), mean corpuscular volume (MCV), mean corpuscular hemoglobin (MCH), mean corpuscular hemoglobin concentration (MCHC) and platelet count (PLT) (flow cytometry based on laser light scatter) (ADVIA 2120, Siemens Healthcare Diagnostics, Tarrytown, USA). The following biochemical parameters were determined: activity of aspartate aminotransferase (AST), creatine kinase (CK), lactate dehydrogenase (LDH) and the concentrations of triglycerides (TG), cholesterol (CHOL), glucose (GLU), total protein (TP), albumin (ALB), urea (UREA) and creatinine (CREA) (Cormay ACCENT 200 Automatic Biochemical Analyzer and Cormay diagnostic kits, Lomianki, Poland).

### Statistical analysis

The normality of parameters distribution and their homogeneity of variance were tested using the Shapiro-Wilk and Levene’s tests, respectively. As none of the data followed a normal distribution, the Kruskall – Wallis test followed by Mann-Whitney U tests with adjustment for multiple comparisons were used for group comparisons at each sampling point. Changes over time were tested using repeated measures Friedman ANOVA. Data are presented as mean ± sem and mean-fold change. Two levels of significance were adopted: * *p* < 0.05 and ** *p* < 0.01.

## Results

**Fig. 1:**
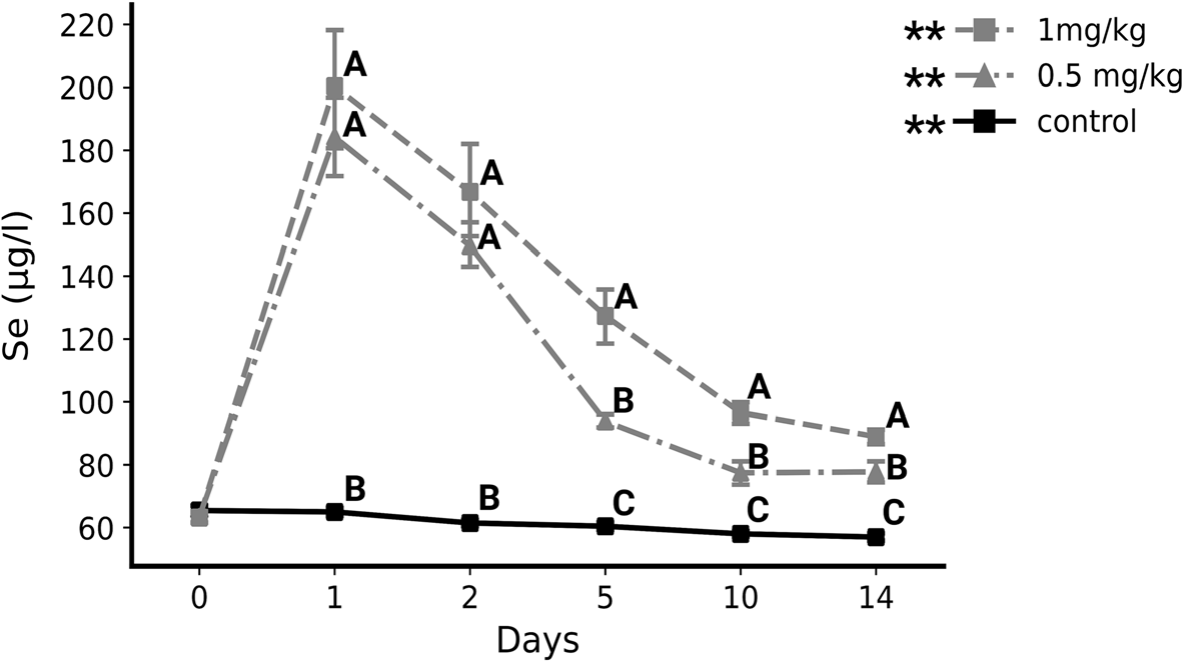
Serum selenium concentration (ug/l) in supplemented and un-supplemented calves (mean + sem). **- *P* < 0.01: Representing the significance of change over time in selenium serum concentration in each studied group (Friedmans ANOVA). Different letters (A, B, C) represent the significant difference (*P* < 0.05) in concentration between groups at each sampling date

**Fig. 2:**
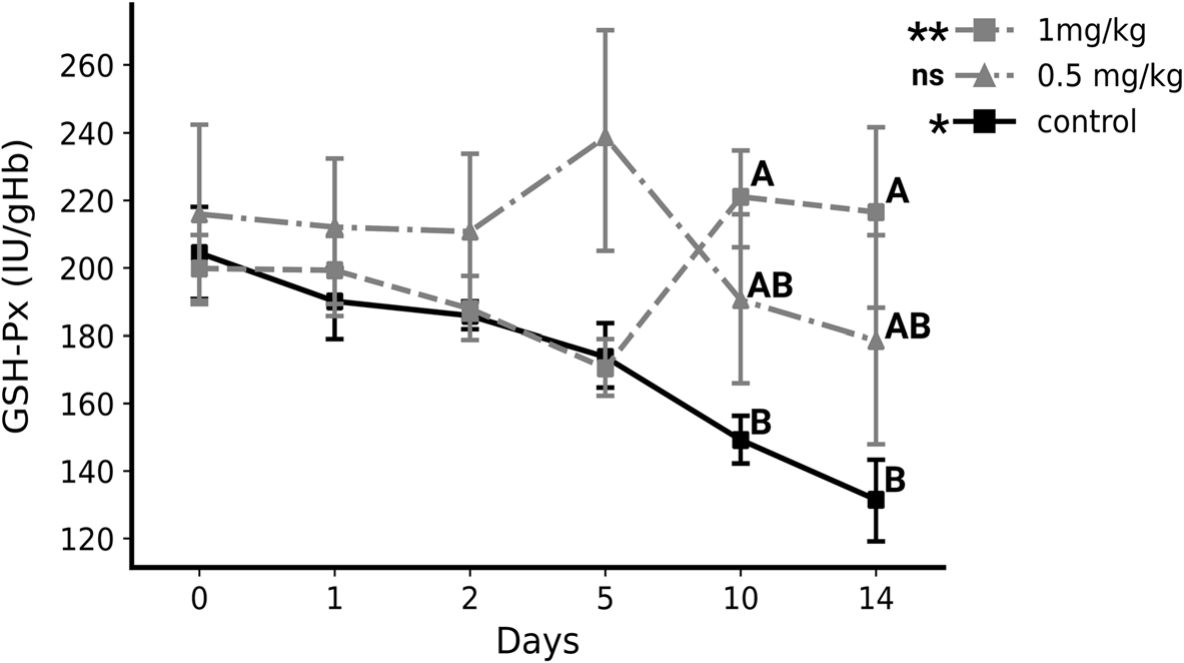
The activity of glutathione peroxidase in supplemented and un-supplemented calves (mean + sem). ns- *P* > 0.05, *- *P* < 0.05, **- *P* < 0.01: Representing the significance of change over time in glutathione peroxidase activity in each studied group (Friedmans ANOVA). Different letters (A, B) represent the significant difference (*P* < 0.05) in glutathione peroxidase activity between groups at each sampling date

The concentration of serum Se increased after supplementation 2.9-fold (from 63.4 ± 1.84 µg/l on day 0 to 184.22 ± 13.73 µg/l on day 1) and 3.17-fold (from 63.17 ± 1.72 µg/l on day 0 to 200.33 ± 21.42 µg/l on day 1) in the 0.5 mg/kg (*P* < 0.01) and 1 mg/kg (*P* < 0.01) groups, respectively, while in the control group concentrations gradually decreased over time (from 65.43 ± 1.63 µg/l on day 0 to 56.97 ± 1.23 µg/l on day 14, *P* < 0.01). Selenium concentrations were higher throughout the experiment (after day 0) in the supplemented groups compared to the control group (*P* < 0.05). In addition, Se concentrations were higher in the 1 mg/kg group compared to the 0.5 mg/kg group from the fifth (1.36-fold, *P* < 0.05) to the 14th day (1.14-fold, *P* < 0.05) (Fig. 1). Over time, GSH-Px activity decreased (from 204.4 ± 14.39 IU/gHb on day 0 to 131.47 ± 12.97 IU/gHb on day 14, *P* < 0.01) in the control group while it increased in the 1 mg/kg group (from 199.83 ± 11.38 IU/gHb on day 0 to 216.53 ± 27.52 IU/gHb on day 14, *P* < 0.05). GSH-Pxwas higher in the 1 mg/kg group compared to the control group on day 10 (1.48-fold, 221.06 ± 15.39 IU/gHb vs. 149.18 ± 7.72 IU/gHb, *P* < 0.05) and day 14 (1.65-fold, 216.53 ± 27.52 IU/gHb vs. 131.47 ± 12.97 IU/gHb, *P* < 0.05) (Fig. 2).

There were no significant differences in any of the haematological parameters between the groups of calves, except on day 14, when the MCV of control calves was, on average, higher than that of the 0.5 mg group, though the absolute difference was small (1.45 pg) (Table 1). Over time, there was a significant decrease in MCV and a significant increase in the number of platelets; the other parameters did not change significantly (Table 1). Significant differences between groups in the concentration of cholesterol (day10; control higher than 0.5 mg), in urea level (day 1; 1 mg higher than 0.5 mg) and in creatinine level (day 0; 1 mg higher than 0.5 mg) were observed. However, values remained within the physiological limits. There were no significant differences in any group of the calves’ in other biochemical parameters and no changes over time in all parameters (Table 2).

**Table 1:** Hematological parameters (mean + sem) in calves

	Group	N	0	1	2	5	10	14
WBC (10^9^/L)	control	6	6.38 ± 0.51	7.11 ± 0.54	7.05 ± 0.65	6.35 ± 0.82	7.19 ± 0.43	6.13 ± 0.35
	0.5 mg/kg	6	6.38 ± 0.85	6.42 ± 0.97	6.07 ± 0.99	7.85 ± 0.73	6.85 ± 0.63	6.84 ± 0.74
	1 mg/kg	6	6.55 ± 0.43	6.8 ± 0.92	6.53 ± 0.8	7.03 ± 0.88	7.31 ± 0.54	6.76 ± 0.64
RBC (10¹²/L)	control	6	7.63 ± 0.6	7.47 ± 0.5	7.35 ± 0.63	7.61 ± 0.45	7.01 ± 0.39	6.99 ± 0.58
	0.5 mg/kg	6	7.23 ± 0.47	7.02 ± 0.32	7.68 ± 0.69	7.69 ± 0.69	7.57 ± 0.6	7.61 ± 0.54
	1 mg/kg	6	7.81 ± 0.5	7.39 ± 0.41	7.6 ± 0.32	8.46 ± 0.63	8 ± 0.43	8.02 ± 0.51
HGB (g/dL)	Control	6	8.73 ± 0.61	8.95 ± 0.97	9.07 ± 0.95	7.57 ± 1	8.67 ± 0.86	8.97 ± 0.87
	0.5 mg/kg	6	7.72 ± 0.75	7.65 ± 0.77	8.12 ± 1.06	7.85 ± 1.02	7.25 ± 0.9	7.43 ± 0.97
	1 mg/kg	6	8.93 ± 0.71	8.33 ± 0.6	8.5 ± 0.51	9.45 ± 0.91	8.6 ± 0.67	8.55 ± 0.88
HCT (L/L)	control	6	32.08 ± 1.75	31.37 ± 1.04	31.2 ± 0.83	31.62 ± 0.95	32.43 ± 1.56	32.52 ± 1.85
	0.5 mg/kg	6	28.62 ± 2.47	27.73 ± 1.93	30.23 ± 3.55	29.72 ± 3.48	27.45 ± 3.24	27.2 ± 3.32
	1 mg/kg	6	32.67 ± 2.51	30.7 ± 2.19	31.28 ± 1.83	35.83 ± 3.65	32.13 ± 2.39	30.37 ± 2.64
MCV (fL)	Control**	6	38.9 ± 1.19	37.17 ± 1.1	35.37 ± 1.35	36.87 ± 1.1	35.13 ± 1.14	34.02 ± 1.29
	0.5 mg/kg^*^	6	39.33 ± 1.33	39.35 ± 1.34	39.98 ± 1.38	37.22 ± 1.34	36 ± 1.65	35.08 ± 1.89
	1 mg/kg^**^	6	40.73 ± 1.01	40.38 ± 0.95	40.05 ± 0.9	39 ± 1.22	39.72 ± 0.87	37.63 ± 1.15
MCH (pg)	control	6	12.07 ± 0.68	11.73 ± 0.65	11.65 ± 0.67	11.58 ± 0.52	11.38 ± 0.44	**11 ± 0.47**^** A**^
	0.5 mg/kg	6	10.57 ± 0.43	10.77 ± 0.68	10.42 ± 0.51	10.05 ± 0.48	10.57 ± 0.5	**9.55 ± 0.61**^**B**^
	1 mg/kg	6	11.38 ± 0.36	11.22 ± 0.34	11.12 ± 0.31	11.1 ± 0.31	11.23 ± 0.28	**10.57 ± 0.55**^**AB**^
MCHC (g/dL)	control	6	27.18 ± 0.29	27.93 ± 0.69	27.45 ± 0.45	27.25 ± 0.58	27.05 ± 0.38	26.97 ± 0.7
	0.5 mg/kg	6	26.88 ± 0.29	27.27 ± 0.94	26.65 ± 0.42	26.22 ± 0.39	26.7 ± 0.29	27.13 ± 0.49
	1 mg/kg	6	27.32 ± 0.41	27.1 ± 0.41	27.08 ± 0.34	26.42 ± 0.24	26.97 ± 0.23	28 ± 0.69
PLT (10^9^/L)	Control*	6	533.17 ± 69.4	653 ± 74.43	840.83 ± 42.06	826.83 ± 68.33	851.5 ± 50.76	737.17 ± 34.63
	0.5 mg/kg*	6	537.17 ± 51.05	672.83 ± 45.89	888.17 ± 40.02	775 ± 100.1	718 ± 86.31	685.67 ± 77.2
	1 mg/kg^*^	6	464.5 ± 51.75	595.33 ± 71.16	744.5 ± 81.31	707.67 ± 93.61	790 ± 63.31	692.17 ± 68.17

Hematological parameters in calves either supplemented with selenitetriglycerides or not supplemented (control) on day 0 (before supplementation) and days 1–14 after supplementation. *- *P* < 0.05, **- *P* < 0.01: Representing the significance of change over time in studied parameters in each studied group (Friedmans ANOVA). Different letters (A, B) represent the significant difference (*P* < 0.05) in concentration between groups in each sampling date

**Table 2:** Biochemical parameters (mean + sem) in calves

	Group	0	1	2	5	10	14
AST (U/l)	control	58.83 ± 2.8	59 ± 2.42	56.67 ± 2.38	56.17 ± 2.4	56.33 ± 2.51	55 ± 3.28
	0.5 mg/kg	60.17 ± 3.75	51 ± 7.13	56 ± 4.5	57.83 ± 0.4	56.17 ± 1.54	55.5 ± 2.58
	1 mg/kg	61.83 ± 3.83	57.5 ± 0.5	54.17 ± 2.46	60.5 ± 0.99	62.5 ± 4.72	55.17 ± 2.83
LDH (U/l)	control	1159.83 ± 66.26	1217.33 ± 90.77	1148.5 ± 58.26	1140 ± 75.18	1165.17 ± 71.55	1117.33 ± 75.07
	0.5 mg/kg	1154.83 ± 95.05	1108.67 ± 93.88	1110.5 ± 107.98	1017.83 ± 85.1	1048.5 ± 71.01	1168.33 ± 97.59
	1 mg/kg	1180.67 ± 24.07	1226.67 ± 34.88	1157.67 ± 34.42	1093 ± 65.04	1179.5 ± 132.76	1291.33 ± 76.73
CK (U/l)	Control	88.43 ± 11.96	94.27 ± 9.57	113.65 ± 16.11	100.42 ± 8.41	143.48 ± 10.7	122.33 ± 11.37
	0.5 mg/kg	107.12 ± 29.22	182.13 ± 86.75	131.73 ± 25.42	100.2 ± 9.7	184.9 ± 34.76	149.78 ± 12.96
	1 mg/kg	103.28 ± 17.96	79.33 ± 13.38	98.77 ± 24.75	99.82 ± 14.27	127.03 ± 6.45	169.37 ± 22.36
TG (mmol/l)	control	0.18 ± 0.03	0.25 ± 0.03	0.24 ± 0.03	0.23 ± 0.03	0.15 ± 0.01	0.22 ± 0.02
	0.5 mg/kg	0.16 ± 0.02	0.19 ± 0.02	0.24 ± 0.03	0.18 ± 0.04	0.2 ± 0.03	0.2 ± 0.03
	1 mg/kg	0.42 ± 0.28	0.2 ± 0.04	0.26 ± 0.04	0.24 ± 0.02	0.16 ± 0.02	0.23 ± 0.04
Chol (mmol/l)	control	1.66 ± 0.15	1.9 ± 0.09	1.76 ± 0.06	1.42 ± 0.1	**2.12 ± 0.11**^** A**^	2 ± 0.17
	0.5 mg/kg	1.9 ± 0.2	1.88 ± 0.17	1.83 ± 0.1	1.55 ± 0.13	**1.67 ± 0.17**^**B**^	1.97 ± 0.27
	1 mg/kg	1.54 ± 0.09	1.55 ± 0.15	1.71 ± 0.17	1.78 ± 0.12	**1.78 ± 0.12**^**AB**^	1.72 ± 0.23
Gluc (mmol/l)	control	6.21 ± 0.24	6.3 ± 0.35	5.91 ± 0.29	6.07 ± 0.33	5.96 ± 0.29	5.72 ± 0.29
	0.5 mg/kg	5.79 ± 0.41	6.55 ± 0.46	5.73 ± 0.06	5.68 ± 0.29	5.26 ± 0.3	5.39 ± 0.22
	1 mg/kg	6.79 ± 0.25	6.93 ± 0.52	6.64 ± 0.3	6.26 ± 0.32	5.28 ± 0.41	5.58 ± 0.35
ALB (g/l)	control	32.87 ± 0.93	33.5 ± 0.82	34.55 ± 0.98	34.73 ± 1.41	33.8 ± 0.74	34.57 ± 0.96
	0.5 mg/kg	31.63 ± 0.63	31.02 ± 0.63	32.03 ± 0.96	32.13 ± 1.09	32.35 ± 0.61	33.02 ± 0.7
	1 mg/kg	32.55 ± 1.16	33.45 ± 0.66	33.47 ± 0.51	33.83 ± 1.01	33.72 ± 0.96	35.33 ± 0.67
TP (g/l)	control	55.73 ± 1.91	48.1 ± 8.5	56.95 ± 1.47	55.75 ± 1.96	53.28 ± 2.34	51.9 ± 1.66
	0.5 mg/kg	56.05 ± 1.22	53.07 ± 1.43	55.05 ± 2.22	51.23 ± 2.17	48.63 ± 1.08	49.22 ± 1.71
	1 mg/kg	56.77 ± 2.32	55.02 ± 1.73	55.12 ± 1.65	56.8 ± 1.85	51.52 ± 2.07	51.68 ± 2.01
Urea (mmol/l)	control	4.28 ± 0.47	**4.15 ± 0.46**^**AB**^	4.26 ± 0.45	4.51 ± 0.18	4.52 ± 0.36	4.51 ± 0.34
	0.5 mg/kg	3.98 ± 0.37	**3.95 ± 0.21**^** A**^	4.25 ± 0.25	4.87 ± 0.34	4.6 ± 0.46	3.79 ± 0.51
	1 mg/kg	4.8 ± 0.48	**4.58 ± 0.58**^**B**^	4.71 ± 0.28	6.8 ± 1.87	5.27 ± 0.25	4.27 ± 0.43
Crea (µmol/l)	control	**83.32 ± 4.12**^**AB**^	89.9 ± 4.08	90.28 ± 8.14	91.08 ± 5.85	93.58 ± 8.02	94.85 ± 11.69
	0.5 mg/kg	**77.52 ± 2.61**^** A**^	80.5 ± 3.18	84.5 ± 5.43	87.05 ± 5.03	84.9 ± 4.29	90.52 ± 6.15
	1 mg/kg	**90.68 ± 3.3**^**B**^	92.13 ± 1.87	96.42 ± 3.45	133.62 ± 29.65	100.6 ± 4.53	92.5 ± 3.35

The activity of aspartate aminotransferase (AST), lactate dehydrogenase (LDH), creatine kinase (CK), and serum concentrations of glucose (Gluc), total protein (TP), albumin (ALB), triglycerides (TG), cholesterol (Chol), urea (Urea), creatinine (Crea) in calves either supplemented with selenitetriglycerides at two doses 0.5 or 1.0 mg/kg or not supplemented (control) on day 0 (before supplementation) and days 1–14 after supplementation. Different letters (A, B) represent the significant difference (*P* < 0.05) in concentration between groups in each sampling date

## Discussion

This is the first study to investigate the effects of selenitetriglycerides in calves. Such basic studies on the biological activity of these products are needed before studies on clinical efficacy to determine whether toxicity is an issue and whether they produce changes in selenium status and related haematological and biochemical parameters beneficial in managing clinical Se deficiency.

The present study showed a rapid increase in the serum Se concentration after a single oral dose of selenitetriglycerides in calves. Selenium concentration increased more than three-fold in both supplemented groups within 24 h, the increase being higher at the 1 mg/kg dose. This supports the hypothesis that using a form with contains more selenium has excellent bioavailability after *per os* administration in unweaned calves. Though comparable studies with this form of Se are not available in calves, previous studies using a single intramuscular injection of sodium selenite (0.1 mg/kg BW) increased serum Se concentration three days after supplementation in calves [10], while dietary sodium selenite supplementation (100 mg/cow/day, cir. 0.17 mg/kg BW) increased cows serum Se within two to six days [11].

In addition to this rapid increase in serum Se concentration, a single oral dose of selenitetriglycerides maintained serum Se concentrations above those in the control group and above baseline concentrations throughout the experimental period (14 days). These results differ from those obtained in serum and liver Se concentrations following a single oral dose of selenium in cattle, where such changes were not observed [12]. Studies performed by Pavlata et al. [13] showed an increase in selenium in the blood of calves (from 70.25 to 127.5 µg/l) after the daily oral administration of selenium yeast (0.6 mg/kg BW) for two months. The single selenitetriglycerides oral doses used in the present study resulted in a larger increase in selenium concentration than the cited studies, albeit for a shorter period. This response in calves confirms the results of previous research performed in dairy cows, which showed a large increase in serum selenium concentration (from 64.92 µg/l to 127.95 µg/l) on the second day after oral administration of selenitetriglycerides [14].

In contrast to the supplemented calves in the present study, serum Se concentrations declined significantly in control calves over time. By the end of the experiment (day 14) the mean serum Se concentration in the control group (56 µg/l) was below the normal range for calves [15]. This decline in serum Se concentration reflected the inadequacy of the Se content/bioavailability of the whole milk and starter feed intake to maintain calf Se status as Se requirements increase with calf growth.

Oral dosing of calves with 1 mg/kg BW of Se resulted in a significant increase in GSH-Px activity between day 0 and day 10. There was also a numeric increase in GSH-Px activity on day 5 in calves administered 0.5 mg/kg BW of Se. These findings indicate that the higher dose is required to improve Se status for longer. Numerous studies confirm response lagging GSH-Px activity (approx. 10–12 days) after Se administration [10, 16, 17]. According to Philipoo et al. [18], the time that elapses from selenium supplementation to the increase in GSH-Px activity results from the fact that selenium is first used to replenish tissue reserves and only then to synthesize peroxidase. On the other hand, Arthur [19] explained this period by the mechanisms of selenium incorporation into erythrocytes during erythropoiesis and the time necessary for the biosynthesis of the enzyme itself. It should be noted that during the monitoring period of this study (0–14 days of age) foetal erythrocytes comprised the majority of the calves’ red cell population, being replaced by adult erythrocytes by eight to 13 weeks of age [20]. Though it should be recognised that this conversion from foetal to adult haemoglobin is a dynamic process which would have already commenced during the monitored response period of the study.

The increase in GSH-Px activity found here after selenitetriglycerides administration is congruent with findings from studies conducted in humans [8] and mice [7] but conflicts with results from studies in sheep. There was no increase in plasma glutathione peroxidase, cytosolic glutathione peroxidase, type I and type II iodothyronine deiodinases and thioredoxin reductase in the brain, adrenal glands, kidneys, liver and thyroid of sheep supplemented orally with 60 mg (cir. 1.2 mg/kg BW) of selenitetriglycerides per animal per day for a month [9]. The activity of plasma GSH-Px increased insignificantly up to the 10th day of the experiment (from 67.0 U/l to 115.6 U/l), but by the 14th day of the experiment, it significantly decreased (to 54.1 U/l) and continued to decline until the end of the experiment. Similarly, no significant increase in GSH-Px activity in whole blood was observed during 28 days of oral selenitetriglycerides supplementation in sheep [9]. Though whole blood GSH-Px is a lag phase indicator of improved selenium status (compared to blood selenium concentration), given that neither plasma GSH-Px (rapid bioindicator of improved selenium status) nor whole blood GSH-Px increased significantly over a month indicates that while the response period was adequate [allowing for erythropoiesis (< 7d)], another factor must account for the non-responsive GSH-Px activity. Differences in GSH-Px activity observed between our result and results obtained by these authors may be related to the fact that the described studies were carried out on adult sheep with a fully developed rumen, which could interfere with the absorption of orally administered selenium. In contrast to the supplemented calves, GSH-Px activity declined significantly in control calves, which was also, as expected, correlated with a significant decrease in serum Se.

In both doses, the administration of selenitetriglycerides did not significantly influence the red cell indices, the number of white blood cells, or platelets in calves. Similar results were obtained for the number of RBC and HGB concentration in calves receiving Se in the form of sodium selenate by Shinde et al. [21]. Also, an earlier study by Bednarek et al. [22] found no effect of selenium (and vitamin E) administration on RBC count and HGB concentration in calves. However, administration of Se in the form of sodium selenite at dose of 2.5 mg/kg for 21 days and 0.25 mg/kg for 16 weeks induced acute and chronic selenosis in calves with significant decreased of total number of RBC and WBC as well as HGB concentration [23]. Some age-related physiological changes in the haemogram were observed in the present study, similarly to data obtained by other authors [20]. The reduction in RBC volume observed is related to the maturation of blood cells as the calves get older [24, 25]. The number of PLT increased significantly throughout the experiment in both control and experimental groups. Platelet count increases rapidly in the first days of a calf’s life [24, 26]. For example, Egli and Blum [27] showed a sharp increase in the number of PLT in calves, from the first to the seventh day of their life, which hardly changed until the 84th day.

In summary supplementation with selenitetriglycerides did not influence hematological parameters in calves, only physiological fluctuations in the values of these indcators were observed.

Supplementation with selenitetriglycerides, at either dose, had no significant effect on the biochemical indices monitored in this study. The glucose results were within the reference range for calves [28]. Inorganic Se supplementation similarly did not affect calves’ blood glucose concentration [21] or in adult cattle [29]. Despite this experimental evidence, theoretically, there is a hypothesized effect of Se on glucose metabolism; studies in rats and human revealed that Se might stimulate glucose intake and regulation of metabolic processes such as glycolysis, gluconeogenesis, fatty acid synthesis, or the pentose phosphate pathway [30].

The lack of an effect of Se supplementation on total protein and albumin concentrations in the present study confirms the findings of Shinde et al. [21] in calves. However, a study in goats showed that selenium might stimulate protein biosynthesis, and Se supplementation may increase this blood total protein concentration in ruminants, but such effects were observed during much longer studies – after 160 days of oral supplementation of selenium [31].

While both the results of the present study and those of Singh et al. [32] found no effect of Se supplementation on blood total cholesterol concentration in calves, Shinde et al. [21] found that Se administration increased the concentration of total cholesterol and its HDL (High-density lipoprotein) fraction in calves. The authors explained this observation by the positive effect of increased blood selenium concentration on pancreatic function, which facilitated the absorption and digestion of dietary fat. The failure of Se supplementation to alter physiologically normal serum triglyceride concentrations in the present study confirms previous findings on Se supplementation in calves [21, 33].

An increase in liver enzymes activity, especially AST, is a sensitive indicator of potential selenium poisoning in ruminants [34]. Hence, it may be inferred from Se supplementation’s failure to alter the activity of liver enzymes (AST and LDH) in the present study that selenitetriglycerides, used at these doses did not adversely affect liver function. A similar finding was reported following supplementation of cows with sodium selenate at a dose of 100 mg per animal for 28 days [11]. However, calves are more sensitive to selenium poisoning. Daily supplementation of calves with sodium selenate at 0.25 mg/kg BW resulted in the clinical signs of sub-chronic selenosis after 12 weeks of administration, when blood selenium concentration exceeded 1680 µg/l. [35]. In an experimental study with buffalo calves, adverse effects appeared when the whole blood selenium concentrations increased above 2000 µg/l, with mortality occurring when blood levels exceeded 3400 µg/l [36]. The maximum concentration of serum selenium in the present study was 200 µg/l, well below the values associated with clinical toxicity.

While CK is one of the most sensitive and fastest indicators of changes occurring in the course of NMD in calves [37], in the absence of NMD in the present study, it is not surprising that the presence or absence of Se supplementation did not alter blood CK activity.

The lack of consistent effects of Se supplementation, at the doses used here, on the serum concentrations of urea and creatinine indicates that there was no adverse effect on renal function of a single selenitetriglycerides administration. Similarly, changes in the concentration of urea and creatinine were not observed by Mudgal et al. [38] and Shinde et al. [21] in calves receiving sodium selenate at a dose of 0.3 mg/kg dry matter and by Singh et al. [32] who induced selenosis by ad lib. feeding of selenium enriched (8.54 ppm) wheat straw for three months. In summary, supplementation with selenitetriglycerides, at either dose, had no significant effect on the biochemical indices monitored in this study. There was no effect of supplementation on glucose concentrations, indicators of protein and fat metabolism, liver enzymes and kidney function. Regarding the study limitations, the product used is not currently commercially available. To confirm these preliminary findings, research should be conducted on a larger group of animals and in order to broaden our understanding of the dose-response relationship, use at least three different doses. In addition, studies should be conducted in beef calves and calves should be monitored for a longer period.

## Conclusions

This experiment showed that oral administration of selenitetriglycerides, at either 0.5 or 1 mg/kg BW, is an effective and safe form of selenium supplementation in calves. A single administration of 0.5 and 1 mg/kg BW of selenitetriglycerides significantly increased serum selenium concentration for 14 days. The obtained results showed that the higher dose of selenitetriglycerides is safe and more effective in improving Se status and the monitored biochemical parameters indicate that the supplementation did not have a negative effect on the functioning of the liver and kidneys. Given these results, it is concluded that selenitetriglycerides are safe to use in further research on their role in managing clinical Se deficiency/NMD in calves.

## Data Availability

The datasets used and analyzed during the current study are available from the corresponding author on reasonable request.

## Abbreviations

GSH-Px: Glutathione peroxidase
WBC: White blood cell count
RBC: Red blood cell count
HGB: Hemoglobin concentration
HCT: Hematocrit
MCV: Mean corpuscular volume
MCH: Mean corpuscular hemoglobin
MCHC: Mean corpuscular hemoglobin concentration
PLT: Platelet count
AST: Aspartate aminotransferase
CK: Creatine kinase
LDH: Lactate dehydrogenase
TG: Triglycerides
CHOL: Cholesterol
GLU: Glucose
TP: Total protein
ALB: Albumin
UREA: Urea
CREA: Creatinine
NMD: Nutritional muscular dystrophy
